# Supplementary material for: Social inequality in infant mortality in Angola: Evidence from a population based study
Source: PLoS One. 2020 Oct 22;15(10):e0241049. doi: 10.1371/journal.pone.0241049 (PMC7580929; doi:10.1371/journal.pone.0241049)
Supplement: S1 Table — (DOCX) [file pone.0241049.s001.docx]

| **Provinces** | [**Population**](javascript:sort('tl',7,true)) Projection  2019-07-01 |
| --- | --- |
| [Bengo](javascript:sym(7495)) | 445,700 |
| [Benguela](javascript:sym(7487)) | 2,543,500 |
| [Bié](javascript:sym(7483)) | 1,709,100 |
| [Cabinda](javascript:sym(7494)) | 824,100 |
| [Cuando Cubango](javascript:sym(7498)) (Kuando-Kubango) | 619,800 |
| [Cuanza Norte](javascript:sym(7489)) (Kwanza-Norte) | 510,000 |
| [Cuanza Sul](javascript:sym(7488)) (Kwanza-Sul) | 2,172,200 |
| [Cunene](javascript:sym(7493)) | 1,157,500 |
| [Huambo](javascript:sym(7482)) | 2,389,200 |
| [Huíla](javascript:sym(7486)) | 2,906,800 |
| [Luanda](javascript:sym(7481)) | 8,247,700 |
| [Lunda Norte](javascript:sym(7491)) | 1,001,100 |
| [Lunda Sul](javascript:sym(7496)) | 629,200 |
| [Malanje](javascript:sym(7484)) (Malange) | 1,141,500 |
| [Moxico](javascript:sym(7490)) | 880,500 |
| [Namibe](javascript:sym(7497)) | 588,400 |
| [Uíge](javascript:sym(7485)) | 1,710,800 |
| [Zaire](javascript:sym(7492)) | 698,500 |
